# Case report: Successful percutaneous extracorporeal magnetic levitation ventricular assist device support in a patient with left heart failure due to dilated cardiomyopathy

**DOI:** 10.3389/fcvm.2023.1093794

**Published:** 2023-01-19

**Authors:** Ping Li, Xiaoying Zhang, Shu Chen, Po-lin Hsu, Tingting Wu, Shirui Qian, Wei Su, Guohua Wang, Nianguo Dong

**Affiliations:** ^1^Department of Cardiovascular Surgery, Union Hospital, Tongji Medical College, Huazhong University of Science and Technology, Wuhan, Hubei, China; ^2^magAssist, Inc., Suzhou, Jiangsu, China

**Keywords:** extracorporeal ventricular assist device, maglev, percutaneous mechanical circulatory support, minimally invasive, heart failure

## Abstract

**Introduction:**

Mechanical circulatory support (MCS) can help to maintain hemodynamic stability, improve cardiac function, reduce cardiac load, and is an important method for the treatment of advanced heart failure. However, traditional MCS systems [IABP, Impella, TandemHerat, veno-arterial extracorporeal membrane oxygenation (VA-ECMO)] are associated with limitations including trauma, a high rate of complications (hemolysis, bleeding) and require complex care from nurses.

**Case summary:**

We report a case of left heart failure resulting from dilated cardiomyopathy in a 24 years-old man. A catheter was placed through the right jugular vein and a drainage tube was positioned under ultrasound guidance through the superior vena cava, right atrium, atrial septum, to the left atrium, and returned to the axillary artery using an extracorporeal magnetic levitation ventricular assist device (VAD). The patient was successfully supported for 10 days and bridged to heart transplant.

**Discussion:**

To the best of our knowledge, this is the first report of the use of an extracorporeal magnetic levitation VAD for MCS *via* a percutaneous approach. Our findings support the wider use of this strategy for patients awaiting myocardial recovery or who require heart bridging or transplantation.

## Introduction

In China, common treatments for temporary mechanical circulatory support (MCS) in patients with cardiogenic shock (CS) include intra-aortic balloon pump (IABP) and veno-arterial extracorporeal membrane oxygenation (VA-ECMO). Although the IABP is simple to operate, the results of the SHOCK-II clinical trial showed that IABP had no clear effect on improving survival in patients with CS (1); Similarly, although VA-ECMO increases systemic flow and pressure and reduces venous congestion in pulmonary circulation without unloading the left side of the heart (2), it can lead to major complications including pump thrombosis, bleeding, ischemic limbs, and harlequin syndrome (3). Therefore, more options for MCS and more suitable implantation strategies are needed to improve prognosis in patients with CS.

The extracorporeal magnetic levitation ventricular assist device (VAD) uses a magnetic levitation rotor that can rotate without friction or wear, with less blood stagnation, turbulence or hemolysis, compared with ECMO and almost no mechanical failure (4). In our previous report, we treated a patient who developed CS following coronary artery bypass surgery for 9 days until discharge using magnetic levitation extra-VAD (5). However, this device needs to be inserted by median sternotomy, which is highly invasive and inconvenient for patients. We modified our approach and performed percutaneous left VAD placement through the right internal jugular vein, with cannulation of the axillary artery for 10 days to successfully bridge the patient to heart transplantation. This method was well-tolerated and associated with less trauma and simplified operation compared with CentriMag, which maximizes patient benefits.

## Case report

A 24 years-old man (height: 185 cm; weight: 78 kg) with a history of dilated cardiomyopathy, pulmonary hypertension, severe mitral insufficiency, and moderate tricuspid insufficiency was admitted with a New York Heart Association (NYHA) cardiac function rating of IV and a left ventricular ejection fraction of 20%. He received extensive medical treatment to increase myocardial contractility, reduce pulmonary hypertension and control heart rate. Unfortunately, the patient developed persistent systemic hypoperfusion with CS. He was given temporary left ventricular assist using a magnetic levitation extra-VAD device. Before implantation, ultrasound examination showed left ventricular dilatation (left ventricular end-diastolic diameter 9.5 cm), systolic dysfunction [left ventricular ejection fraction 13%; arterial blood pressure 80/60 mmHg; N-terminal pro-B-type natriuretic peptide (NT-proBNP) 2,910 pg/ml], and pulmonary artery systolic blood pressure of 57 mmHg.

**FIGURE 1:**
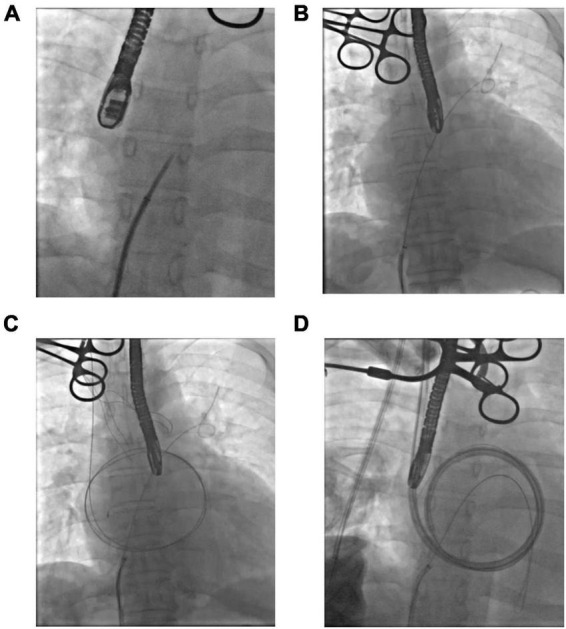
Percutaneous intubation procedure. **(A)** Atrial septal puncture. **(B)** Insertion of the snare. **(C)** Jugular guide wire creates a passage in the left atrium. **(D)** Drainage tube placement.

To circumvent the difficulties of atrial septal puncture through the jugular vein, puncture through the femoral vein was performed. A guide wire was inserted through the right femoral vein puncture to achieve right heart catheterization under the guidance of ultrasound and digital subtraction angiography (DSA) (Figure 1A, and a snare was placed in the right atrium along the femoral vein guide wire (Figure 1B). The catheter was delivered through the right jugular vein, captured by a snare, and placed into the left atrium along the femoral vein guide wire (Figure 1C). A drainage tube was placed using the guide wire approach to establish a drainage path through the jugular vein, superior vena cava, right atrium, and atrial septum to the left atrium (Figure 1D). Finally, the axillary artery was exposed and blocked under the right clavicle, an 8 mm artificial blood vessel was anastomosed end-to-side, and the outflow tube was inserted to establish left atrium-extra-VAD-axillary artery circulation assistance. The pump flow was set at approximately 3 L/min and the speed was 2,500 rpm.

**FIGURE 2:**
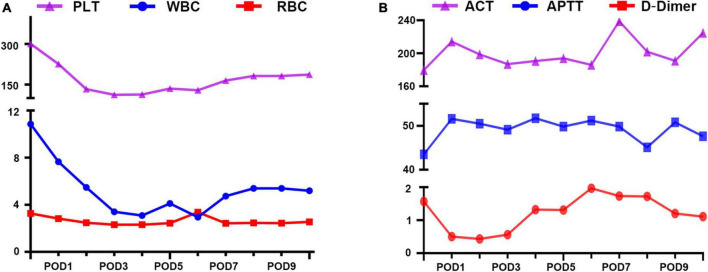
Lab rest results during extra- ventricular assist device (VAD) support. **(A)** Blood compatibility. PLT, platelet; RBC, red blood cell; WBC, white blood cell. **(B)** Anticoagulation management. ACT, active clotting time (s); APTT, activated partial thromboplastin time (s); D-Dimer (mg/L).

Hemodynamic and hematological parameters were monitored throughout extra-VAD implantation, and indicated good recovery. No hemolysis-related complications occurred (Figure 2A), and liver and kidney function were unaffected (Table 1). Heparin was used for anticoagulation therapy to maintain the target activated coagulation time (ACT) between 180 and 220 s and maintain the target activated partial thromboplastin time (APTT) between 40 and 55 s (Figure 2B). High-sensitivity troponin I reached 2,985.1 ng/L postoperatively and rapidly decreased to 647.7 ng/L on the second postoperative day. Similarly, lactic acid levels decreased to 0.6 mmol/L the day after surgery and remained between 0.4 and 1.0 mmol/L thereafter. These results indicated a rapid improvement in cardiac function. The extra-VAD device was removed 10 days later, and ultrasound results showed a left ventricular end-diastolic diameter of 7.6 cm. The patient subsequently underwent a successful heart transplant.

**TABLE 1:** Lab rest results during extra- ventricular assist device (VAD) support.

	POD1	POD2	POD3	POD4	POD5	POD6	POD7	POD8	POD9	POD10
Cr	149.3	103.2	83.1	81.2	79.8	86.6	91.4	104.1	107.4	110.7
BUN	7.28	5.07	2.94	2.5	2.26	3.22	3.36	3.92	4.18	4.5
Bilirubin	13.4	15.3	14.1	12	12.8	10.7	8.9	8.9	10.6	10.6
LAC	2.38	0.6	0.47	0.5	0.45	0.65	0.7	0.75	0.97	0.5
CK	337	666	460	249	–	67	42	–	–	–
LDH	323	354	294	277	296	305	323	331	389	417

BUN, blood urea nitrogen (mmol/L); CK, creatine kinase (U/L); Cr, creatinine (μmol/L); LAC, lactic acid (mmol/L); LDH, lactate dehydrogenase (U/L).

## Discussion

Cardiogenic shock is a recognized cause of death in patients with heart failure due to low cardiac output resulting in severe hypoperfusion of vital organs, with a short-term mortality rate of more than 50% (6). MCS can quickly stabilize hemodynamic parameters, ensure effective perfusion of organs, and improve cardiac function. In recent years, clinicians have increasingly used short-term MCS to improve adverse outcomes in patients with CS (7).

Traditional short-term MCS units such as Impella, TandemHerat, and VA-ECMO use mechanical bearing axial flow pumps. In contrast, the magnetic levitation centrifugal pump increases blood compatibility and reduces the incidence of pump thrombosis, hemolysis, and gastrointestinal bleeding, thereby minimizing blood loss, the need for blood transfusion, and postoperative inflammation (8, 9). In addition, reduced thrombosis minimizes the amount of anticoagulation required to prevent thrombosis; therefore, postoperative anticoagulation management is simpler compared with VA-ECMO.

In addition, only CentriMag can offer full circulatory support; the rest of the device such as Impella CP with up to 4.3 L/min, Impella 5.5 with 5.5 L/min (Axial pump may overestimate the flow rate due to the number are not directly reading from ultrasonic flow sensor) and TandemHeart with 4 L/min can only provide partial support. In life threatening end stage heart failure patient, the support average flow range is from approximately 3.5–6.0 L/min (10). In these cases, impella or TandemHeart may not provide sufficient flow for the patients. Therefore, ECMO and Impella (ECPELLA) are combined to treat cardiogenic shock (11). However, combining two different mechanical circulatory support systems will potentially increase complications.

No bleeding or thrombotic complications and no hemolysis were observed during the entire course of circulatory support, which is consistent with our previous report (5). While our method of extra-VAD is designed for short-term use (≤30 days), we have found it to be well-tolerated over longer periods of time.

In addition, we modified the conventional method of median sternotomy and re-cannulation to a percutaneous approach. To our knowledge, this is the first time this surgical method has been reported in the literature and suggests that minimally invasive, short-term and mid-term external magnetic levitation artificial heart implantation should be considered for widespread use.

Percutaneous left VAD reduces left ventricular volume, wall stress, and myocardial oxygen consumption whilst increasing cardiac output and coronary perfusion to minimize myocardial ischemia and hemodynamic failure in high-risk patients with heart failure. This minimally invasive method greatly reduces patient trauma and avoids the risks associated with complicated thoracotomy (12).

Additionally, the percutaneous approach reduces blood loss and potential coagulation disorders, which simplifies postoperative care (13). With transjugular intubation, patients may walk early after surgery, which can accelerate postoperative activity and rehabilitation and reduce the occurrence of postoperative bed rest complications such as deep vein thrombosis and pulmonary infection (14). In addition to clinical benefits, this minimally invasive approach can significantly shorten the length of hospital stay, restore cardiac function more rapidly and greatly reduce medical costs compared with established approaches (15).

In conclusion, we propose a novel, minimally invasive and easy-to-care mode of extracorporeal ventricular assistance using an extracorporeal magnetic levitation VAD *via* jugular vein intubation. This model avoids unnecessary surgeries and is associated with fewer complications than existing methodologies. Further clinical data are necessary to evaluate the advantages of this model over traditional MCS strategies.

## Data availability statement

The original contributions presented in this study are included in this article/supplementary material, further inquiries can be directed to the corresponding author.

## Ethics statement

The studies involving human participants were reviewed and approved by Union Hospital, Tongji Medical College, Huazhong University of Science and Technology. The patients/participants provided their written informed consent to participate in this study.

## Author contributions

PL: data curation, formal analysis, methodology, software, validation, visualization, and writing—original draft. XZ: methodology, investigation, validation, software, and formal analysis. SC and P-LH: methodology, validation, and writing—review and editing. TW and SQ: writing—review and editing. WS and GW: resources and supervision. ND: conceptualization, review and editing, supervision, and funding acquisition. All authors reviewed the manuscript and approved the submitted version.

## References

[1] Thiele H Schuler G Neumann F Hausleiter J Olbrich H Schwarz B Intraaortic balloon counterpulsation in acute myocardial infarction complicated by cardiogenic shock: design and rationale of the intraaortic balloon pump in cardiogenic shock II (IABP-SHOCK II) trial. *Am Heart J.* (2015) 169:E3–4. 10.1016/j.ahj.2014.11.001 25819870

[2] Mohite P Sabashnikov A Koch A Binu R Padukone A Kaul S Comparison of temporary ventricular assist devices and extracorporeal life support in post-cardiotomy cardiogenic shock. *Interact Cardiovasc Thorac Surg.* (2018) 27:863–9. 29905854 10.1093/icvts/ivy185

[3] Telukuntla K Estep J. Acute mechanical circulatory support for cardiogenic shock. *Methodist Debakey Cardiovasc J.* (2020) 16:27–35. 10.14797/mdcj-16-1-27 32280415 PMC7137625

[4] Takayama H Soni L Kalesan B Truby L Ota T Cedola S Bridge-to-decision therapy with a continuous-flow external ventricular assist device in refractory cardiogenic shock of various causes. *Circ Heart Fail.* (2014) 7:799–806. 10.1161/CIRCHEARTFAILURE.113.000271 25027874 PMC4369383

[5] Li P Wu L Dong N. First experience of magnetically levitated extracorporeal left ventricular assist device for cardiogenic shock in China. *Esc Heart Fail.* (2022) 9:1471–3. 34981656 10.1002/ehf2.13769 PMC8934984

[6] Jentzer J van Diepen S Barsness G Henry T Menon V Rihal C Cardiogenic shock classification to predict mortality in the cardiac intensive care unit. *J Am Coll Cardiol.* (2019) 74:2117–28. 10.1016/j.jacc.2019.07.077 31548097

[7] Balthazar T Vandenbriele C Verbrugge F Den Uil C Engstroem A Janssens S Managing patients with short-term mechanical circulatory support JACC review topic of the week. *J Am Coll Cardiol.* (2021) 77:1243–56. 10.1016/j.jacc.2020.12.054 33663742

[8] Mehra M Goldstein D Uriel N Cleveland J Yuzefpolskaya M Salerno C Two-year outcomes with a magnetically levitated cardiac pump in heart failure. *N Engl J Med.* (2018) 378:1386–95. 10.1056/NEJMoa1800866 29526139

[9] Mohite P Zych B Popov A Sabashnikov A Saez D Patil N CentriMag (R) short-term ventricular assist as a bridge to solution in patients with advanced heart failure: use beyond 30 days. *Eur J Cardiothorac Surg.* (2013) 44:E310–5. 10.1093/ejcts/ezt415 23990618

[10] John R Long J Massey H Griffith B Sun B Tector A Outcomes of a multicenter trial of the levitronix CentriMag ventricular assist system for short-term circulatory support. *J Thorac Cardiovasc Surg.* (2011) 141:932–9. 10.1016/j.jtcvs.2010.03.046 20605026

[11] Nakamura M Imamura T. Practical management of ECPELLA. *Int Heart J.* (2020) 61:1094–6. 10.1536/ihj.20-172 33116023

[12] Saeed D Maxhera B Kamiya H Lichtenberg A Albert A. Alternative right ventricular assist device implantation technique for patients with perioperative right ventricular failure. *J Thorac Cardiovasc Surg.* (2015) 149:927–32. 10.1016/j.jtcvs.2014.10.104 25433641

[13] Vieira J Ventura H Mehra M. Mechanical circulatory support devices in advanced heart failure: 2020 and beyond. *Prog Cardiovasc Dis.* (2020) 63:630–9. 10.1016/j.pcad.2020.09.003 32971112

[14] Sweity E Alkaissi A Othman W Salahat A. Preoperative incentive spirometry for preventing postoperative pulmonary complications in patients undergoing coronary artery bypass graft surgery: a prospective, randomized controlled trial. *J Cardiothorac Surg.* (2021) 16:241. 10.1186/s13019-021-01628-2 34429138 PMC8383237

[15] Santos P Ricci N Suster E Paisani D Chiavegato L. Effects of early mobilisation in patients after cardiac surgery: a systematic review. *Physiotherapy.* (2017) 103:1–12. 10.1016/j.physio.2016.08.003 27931870

